# Examining Heterogeneity in the Impacts of Socio-Emotional Curricula in Preschool: A Quantile Treatment Effect Approach

**DOI:** 10.3389/fpsyg.2021.624320

**Published:** 2021-10-28

**Authors:** Zhiling Meng Shea, Jade Marcus Jenkins

**Affiliations:** School of Education, University of California, Irvine, Irvine, CA, United States

**Keywords:** socio-emotional development, curriculum intervention, quantile treatment effect, preschool, emotion knowledge, executive function, problem-solving skills

## Abstract

We examine treatment effect heterogeneity using data from the Head Start CARES study, in which a sample of preschool centers was randomly assigned to either one of three curricula interventions targeting socio-emotional (SE) skills (i.e., emotional knowledge, problem-solving skills, and executive functions) or to continue using their “business-as-usual” curriculum. Most existing research estimates only mean differences between treatment and control groups, and uses simple subgroup analyses to assess treatment heterogeneity, which may overlook important variation in treatment effects across the *ex post* outcome distribution. We use quantile treatment effects analyses to understand the impacts of these curricular interventions at various parts of the outcome distribution, from the 1st percentile to the 99th percentile, to understand who benefits most from SE curricula interventions. Results show positive impacts of the curricula interventions on emotional knowledge and problem-solving skills, but not equally across the full skill distribution. Children in the upper half of the emotional knowledge distribution and at the higher end of the problem-solving skills distribution gain more from the curricula. As in the study’s original mean-comparison analyses, we find no impacts on children’s executive function skills at any point in the skills distribution. Our findings add to the growing literature on the differential effects of curricula interventions for preschool programs operating at scale. Importantly, it provides the first evidence for the effects of SE curricula interventions on SE outcomes across children’s outcome skill levels. We discuss implications for early education programs for children with different school readiness skills.

## Introduction

Children who enroll in Head Start programs in the United States are more likely to grow up under conditions of adversity. Poverty often increases children’s exposure to multiple stressors, including family instability, crowded living conditions, and community violence (Evans, 2004; Yoshikawa et al., 2012). Hence, it may be especially important for such children to have targeted support in learning how to understand and manage their feelings, regulate their behaviors, and develop interpersonal relationships that provide emotional support (Denham et al., 2014). These socio-emotional (SE) behaviors are also critical to fostering readiness for self-directed learning (Morris et al., 2013b). Because children living in poverty are more likely to attend lower-quality schools, their SE skills may be even more important for school success compared to their higher-income peers (Yoshikawa et al., 2012). Understanding how children’s SE skills develop during preschool is crucial for helping socioeconomically disadvantaged children gain such skills and avert other developmental risks.

To promote SE skill development for economically disadvantaged children, a large-scale randomized study, the Head Start Classroom-based Approaches and Resources for Emotion and Social skill promotion (HS CARES), was conducted in the United States using three evidence-based curricula (i.e., Preschool PATHS, Incredible Years, and Tools of Mind) (Morris et al., 2014). SE development contains a large set of skills, including emotion knowledge (e.g., the ability to identify positive and negative emotions), social problem-solving skills (e.g., social competence and control of aggressive-oppositional impulses), and executive functioning (EF) skills (Riggs et al., 2006; Ursache et al., 2012; Morris et al., 2013b, 2014; Blewitt et al., 2020). The three HS CARES curricula were shown to boost skills across all SE domains in previous studies (Bierman et al., 2008a; Raver et al., 2011). HS CARES showed that the three curricula improved teachers’ instructional practices, and two of the three curricula (Preschool PATHS and Incredible Years) promoted students’ emotion knowledge and problem-solving skills. Similar results were found when the curricula were implemented in other Head Start studies (e.g., Chicago Preschool Readiness Project and the REDI project). The results suggest that curricula can be effective in improving the SE development of preschool-aged children who are economically disadvantaged.

However, almost all evidence of curricula interventions is derived from mean-oriented analyses, and therefore cannot detect treatment differences at other statistical moments (e.g., median, 90th percentile), particularly in the domain of children’s SE development. Further, most studies have examined treatment effect heterogeneity by examining subgroups defined by a pre-existing characteristic, such as their baseline skills, creating subgroups based on these characteristics, and interacting this subgroup indicator with treatment as a moderator, or estimating treatment effects separately by subgroup. These analyses might miss the changes in children’s achievement that occurred after random assignment, which could potentially lead to a reordering of the outcome scores. A growing literature in economics suggests that focusing exclusively on mean impacts or subgroup analyses potentially conceals important heterogeneities in the impact of an intervention on the distribution of outcomes, and ignores the potential effects of an intervention for particular groups of students with different levels of skills (Bitler et al., 2006).

A recent study examined the distributional impacts of several skill-specific preschool curricula (literacy- and language-focused) on child outcome skills with a quantile treatment effect (QTE) approach using experimental data from the Preschool Curriculum Evaluation Research (PCER) (Nguyen, submitted). The method is distinct from subgroup analyses because it relies on skills *after* the interventions, while baseline subgroup analyses rely on examining groups of children based on their characteristics and skills *before* the intervention begins. The study found significant differential effects for children’s skills at certain points in the outcome distribution: literacy curricula improved letter-word scores for children at the bottom of the distribution and spelling scores for children at the top of the distribution. The results imply that skill-specific curricula could affect children’s skill growth differently for children at different outcome skill levels. Importantly, most mean-oriented and subgroup analyses studies could not detect such effects. Our study builds on this previous literature and examines treatment effect heterogeneity with QTE using data from the HS CARES study, where a sample of preschool centers were randomly assigned to either one of three curricula interventions targeting SE skills or to continue using their “business-as-usual” curriculum. Specifically, we use a QTE approach to examine the effects of the SE curricula interventions across the distributions of children’s outcome SE skills.

We test three hypotheses concerning which types of children are most likely to benefit from the curricula interventions. The first is the “compensatory” hypothesis, which predicts that skill-specific curricula will boost achievement for children at the bottom of the skills distribution (Rutter, 1987). The second hypothesis is “skills beget skills,” forecasting that the treatment will primarily improve children’s SE development for children in the top tier of the skills distribution (Cunha and Heckman, 2007). The third hypothesis is “Goldilocks,” suggesting that curriculum interventions will be more effective for children somewhere in the middle of the distribution, as opposed to either end (Miller et al., 2016). Given that the goal of the SE curricula in HS CARES is to promote school readiness and reduce achievement disparities for low-income children who may be vulnerable to behavioral problems (Morris et al., 2014), it is important to know how curricula practices affect children with the strongest skills and at the highest part of the outcome distribution (i.e., in the 90th percentile and above), those at the median (i.e., around the 50th percentile), and children who are struggling with certain SE skills in the lower tier of the outcome distribution (i.e., in the 1st to 10th percentiles). Indeed, the training model and organizational focus of the HS CARES curricula may lead coaches, directors, or teachers to devote more or less attention to students with different skill mastery in their attempts to prevent behavioral problems or promote continued growth. Understanding this variation in effects of curricula interventions is critical for suggesting ways to improve the design or implementation of effective programs and services in early education settings (Morris et al., 2013b).

In the following sections, we review the previous literature related to our study. We then describe our methodological design and detailed measures in the methods section. Finally, we report our results and discuss implications for policy and practice.

## Background and Prior Literature

### Socio-Emotional Skills

It is important to acknowledge that the various skills which we generally characterize as SE are not independent from each other. Socio-emotional skills include a broad set of abilities such as the ability to identify positive and negative emotions, social problem-solving (e.g., social competence and control of aggressive-oppositional impulses), and EF skills (e.g., cognitive flexibility, inhibitory control, and working memory) (Riggs et al., 2006; Ursache et al., 2012; Morris et al., 2013b, 2014; Blewitt et al., 2020). Growth in emotion knowledge can promote the capacity to regulate problem behaviors, reduce conflict and aggression, and foster positive teacher-child and peer social relations (Graziano et al., 2007; Raver et al., 2007; Denham et al., 2015). Children who have higher self-regulation skills may form more positive relationships with peers (Choi et al., 2018). Although different scholars may characterize or structure these constructs over developmental stages in different ways, we consider the entire range of SE skills to be important for healthy child development. In our study, we consider emotion knowledge, problem solving, and EF as SE skills, the most debatable of which being EF skills.

We include EF in our study for three reasons. First, EF overlaps with the broader construct of SE skills. SE skills refer to the process of acquiring social and emotional competence, including emotional self-awareness and a wide range of self-regulatory, pro-social, and problem-solving skills, sometimes including EF skills. EF skills can generally be considered as whichever skills are not academic (e.g., math and reading) but are nonetheless important for academic success, much like SE skills (Zelazo et al., 2016). Second, although EF is more clearly defined in neurocognitive and cognitive domains (i.e., inhibitory controls, working memory, and cognitive flexibility), there is no consensus on the construct of EF from educational and developmental perspectives. For example, the terms self-regulation and EF may be used differently in education and psychology research, even though the actual constructs of self-regulation and EF overlap substantially (Morrison and Grammer, 2016). Third, the curricula interventions we examine were primarily focused on promoting children’s SE skills, and EF was also categorized within the SE domain in the three curricula interventions. Specifically, in the original study, SE was defined as the developing capacity of the child to form close and secure adult and peer relationships; experience, regulate, and express emotions (i.e., subgroups of EF skills) in socially and culturally appropriate ways; and explore the environment and learn — all in the context of family, community, and culture.

Sociocultural theory suggests that children’s SE skills are malleable, particularly through curricula interventions that target such skills (Vygotsky, 1978). This theory proposes that development occurs in the context of the interactions between children and their social environment. This includes interactions with adults as well as peers. The theory considers language as a critical tool for the formation of thought. Initially, these tools are external, such as a picture or language spoken aloud, but in time they become automatized and internalized as children remember the significance of a picture or engage in internal self-talk to help regulate their own emotions and learning. In SE curricula interventions, teachers are trained to use activities that embody these developmental ideas and specifically aim at improving emotions and executive functions. For example, the Tools of Mind curriculum promotes children’s intentional and self-regulated learning through structured “make-believe,” or “pretend,” play activities (Friedman-Krauss et al., 2017).

Early enrichments that promote lasting gains in SE development may contribute in critical ways to positive school adaptation and long-term well-being (Heckman, 2006; Heckman et al., 2013). This is because SE development underlies children’s behaviors, especially in two areas considered to be central to longer-term success: learning behaviors, which refer to children’s ability to focus their attention and behavior during classroom activities, and social behaviors, which are children’s positive interactions with peers and teachers (Morris et al., 2014). Emotion regulation, for instance, could improve the ability to focus selective attention and apply the mental processes necessary for learning (Blair, 2002); preschoolers’ problem-solving skills, such as competent or aggressive behavioral responses to peers, are associated with children’s social skills and could improve school success (Denham and Bouril, 1994). The literature further suggests that children who are emotionally well-regulated may be better able to elicit behavior from others that promotes school-based learning and are more likely to be perceived by their teachers as attentive and cognitively advanced during instruction (Denham et al., 2003). Further, correlational studies suggest that children with higher executive function skills in preschool have better educational outcomes in elementary and high school (Clark et al., 2010; Best et al., 2011; Becker et al., 2014; Nguyen and Duncan, 2019). Early SE skills are also likely to be particularly crucial for low-income children (Dodge et al., 2015; Diamond, 2016). Note that the research linking SE skills and later well-being are not causal and thus may overstate their impacts on later outcomes. However, there exists enough evidence and theoretical grounding to support the idea that SE skills likely play an integral role in fostering children’s well-being.

### Curricula Interventions Promoting Socio-Emotional Skills

Most curricula programs aim to strengthen teachers’ capacities to foster children’s SE development, and research efforts have focused on understanding the impact both on child outcomes (Domitrovich et al., 2007; Bierman et al., 2014; Morris et al., 2014; Jones et al., 2015; Werner et al., 2016) and on teaching quality (Morris et al., 2014; Blewitt et al., 2020). The curricula interventions included in Head Start CARES provided training and coaching, in which playful and guided activities are strategically organized to present children with learning opportunities that are intended to be focused, sequential, and cumulative. Prior empirical evidence had shown that they were efficacious with low-income children, specifically in Head Start (Raver et al., 2011; Bierman et al., 2014; Morris et al., 2014). Bierman et al. (2014) created the Research Based Developmentally Informed (REDI) intervention that was designed as an enrichment program to be integrated into the existing framework of Head Start classrooms, focusing primarily on SE skills using the PATHS Preschool curriculum and language/emergent literacy skill development. It includes lesson plans, center-based extension activities, and training and weekly classroom coaching in “teaching strategies” to use throughout the day. After the intervention, children in REDI classrooms improved their phonemic decoding skills, learning engagement, and competent social problem-solving skills, and reduced problem behaviors compared with children in the control group.

Another curricula intervention targeting SE skills designed for low-income children is the Chicago School Readiness Project (CSRP). The program originated in 2003 and randomly assigned Chicago Head Start centers to a set of classroom-based interventions that included teacher training in classroom behavior management and mental health consultation. The CSRP increased preschool students’ levels of attention and executive function, and improved children’s early verbal, math, and behavioral-emotional skills, including reduced impulsivity (Raver et al., 2011; Morris et al., 2013a; Watts et al., 2018; McCoy et al., 2019).

The HS CARES demonstration study was the largest comprehensive study of SE curricula intervention to improve SE skills, conducted in 104 Head Start centers with three SE enhancements: Preschool PATHS (PATHS), Incredible Years (IY), and Tools of Mind (Tools) (Morris et al., 2014). PATHS focuses on training teachers to use clearly outlined weekly lessons and teaching strategies to improve children’s knowledge of emotions and social problem-solving skills, including the ability to recognize, understand, and communicate about emotions; interpret difficult social situations; and select from a set of various competent solutions for such situations. The IY Teacher Training Program focuses on training teachers to create an organized classroom climate that supports children’s behavior regulation in the context of positive teacher–child relationships. Tools is a 1-year adaptation of the curriculum’s typical 2-year length, training teachers to support children’s planning and enacting “make-believe” (or “pretend”) play and role-playing games to strengthen children’s ability to regulate their emotions and behavior. A central component of Tools is a daily 50-min period devoted to adult-supported pretend play. This component is organized and scaffolded by teachers to enhance children’s ability to plan for and understand various social roles — such as the role of family members — while strengthening their memory, improving their ability to focus their attention, and understanding both their own and their peers’ perspectives.

Each curriculum intervention targets a different set of skills. For example, the primary targeted PATHS outcomes include children’s skills in emotion knowledge, social problem-solving skills, and social behaviors, while the secondary PATHS targeted outcomes include children’s skills in executive function, behavior regulation, and learning behaviors. IY targets children’s outcomes primarily in executive function, behavior regulation, and learning behaviors, and secondarily in emotion knowledge, social problem-solving, and social behavior skills. Tools improves children’s skills primarily in executive function and learning behaviors, and secondarily in behavior regulation, emotion knowledge, social problem-solving skills, as well as learning behaviors. Although there are differences among the three interventions in targeting skills, we consider all three curricula interventions as a whole because they are interrelated in improving a broad set of children’s SE skills (e.g., executive function, emotion knowledge, and social problem-solving skills).

The study showed that PATHS and IY improved student emotion knowledge, social problem-solving skills, and social behaviors. Tools produced positive impacts on emotion knowledge but did not increase executive function skills. All interventions improved teachers’ instructional practices. The study also examined the effects of each intervention by children’s initial level of behavior problems at the beginning of the year and by child gender. The only significant differential impacts of the curricula were for children with high initial levels of behavioral problems in IY having fewer behavioral problems at the end of treatment, and that IY produced greater impacts for boys (marginally significant) on executive function and social problem-solving skills (i.e., challenging situations competent response). Tools also showed stronger positive impacts in social problem-solving skills for boys. Importantly, the HS CARES impact analysis study did not examine differences in the effects of the curricula at different points in the distribution of children’s SE outcome measures, which is the focus of our study.

Although all studies discussed above have demonstrated positive impacts on children’s SE development, some studies have found that SE curricula interventions do not affect child outcomes. For example, a systematic review including six experimental studies examined the effectiveness of the Tools curriculum, finding that the curriculum only improved math skills and not a range of SE skills (Baron et al., 2017). But it is also possible that null mean impacts are hiding different effects across the distribution of outcomes (Bitler et al., 2015). Thus, there is no clear consensus regarding the effects of SE curricula on children’s socio-emotional development.

### Methods to Assess Treatment Heterogeneity

There are different methods to unpack treatment effect heterogeneity in educational interventions (Schochet et al., 2014). In our study, we estimate whether treatment effects vary along the distribution of children’s SE outcome measures, referred to as distributional analyses. Most studies examine treatment effect heterogeneity by examining subgroups defined by a pre-existing characteristic, such as their baseline skills, creating subgroups based on these characteristics, and interacting this subgroup indicator with treatment as a moderator, or estimating treatment effects separately by subgroup. The distinction between the effects captured by these baseline subgroup analyses and distributional analyses is that the subgroup analyses rely on examining groups of children based on their characteristics and skills *before* the intervention begins, while the distributional analyses can rely on skills *after* the intervention. Whereas the moderation analyses focus on mean comparisons based on subgroups of children, the distributional analyses make non-mean-based comparisons.

In this study, we examine the degree to which the effects of SE curricula vary across the *ex post* measures of achievement, taking advantage of randomized assignment to treatment (skill-specific curricula) and control (business-as-usual curricula) centers to estimate QTE using the outcome measures taken at the end of the intervention (Firpo, 2007). QTEs analyses allow us to understand the effects at various parts of the distribution, including the upper and lower tails, which could have important implications for understanding who benefits most from an intervention. This is distinct from regression, which assumes a uniform, constant effect of the intervention for all participants (Schochet et al., 2014). The essence of QTE is generating treatment and control differences at each point in the outcome distribution, in contrast to baseline moderation analyses, which compare the mean outcomes for children belonging to different subgroups determined *ex ante*. This is important because, by only providing estimates of how the explanatory variables impact the mean of the outcome variable, traditional subgroup analyses might overlook important variations in differences across other parts of the outcome distribution. This method has been used in other studies of educational interventions (e.g., Neal and Schanzenbach, 2010; Angrist et al., 2012; Jackson and Page, 2013), and a few recent studies have used QTE to evaluate the effects of early childhood educational interventions, showing that they in fact do not have a uniform effect on all program recipients (Bitler et al., 2014; Nguyen, submitted).

The results from estimating variation in treatment effects across the distribution of a post skill measure might seem comparable to examining variation in average treatment effects for subgroups of children based on their pretest scores, but these two analyses could produce different findings (Schochet et al., 2014). First, the estimates from two methods might differ if there were changes in children’s achievement that occurred after random assignment (i.e., after the pretest was administered) which could affect those with high and low pretest scores. For instance, the quality of the classroom instruction or the implementation of the curriculum intervention could vary across the sample. This could potentially lead to a reordering of the posttest scores that would be captured in the QTE analyses but not in the subgroup analyses. Second, if an intervention has large positive effects for children with the lowest baseline scores and large negative effects for children with high baseline scores, then the distribution of the outcome variable could be reversed. In this case, the baseline subgroup analyses will show positive impacts for children scoring low on the outcome and negative impacts for children high on the outcome, but the QTEs will show small impacts on any percentile of the outcome distribution. Most importantly, for many policy interventions, including Head Start, intervention effects on the (*ex post*) distribution are of substantial interest, and the rhetoric about closing achievement gaps is about *ex post* (of some reform or treatment) scores, not *ex ante* ones (Bitler et al., 2014). Using QTE precisely captures these *ex post* effects, while linear regression results for subgroup analysis based on baseline skills capture the *ex ante* effects. Given the number of null findings from the HS CARES study, and their aim to reduce gaps in SE skills, further probing these impacts with QTE is an important next step.

Indeed, recent studies of educational interventions using QTE have revealed these types of nuanced results. For example, Bitler et al. (2014) used QTE to examine the effects of Head Start attendance on student cognitive and non-cognitive outcomes, finding that random assignment to Head Start led to large and statistically significant gains in cognitive achievement, which were largest at the bottom of the skills distribution, specifically for children at the 1–3, 13, and 43 percentiles. In their study, baseline scores were also imbalanced and missing, making them unreliable for subgroup analyses. Similarly, Nguyen (submitted) examined the effects of literacy curricula compared with locally developed curricula on children’s cognitive development and found that children at the bottom of the distribution of letter-word knowledge (between the 9th and 33rd percentiles) and at the top of the distribution of spelling scores (between the 71st and 94th percentiles) benefited the most from the literacy curricula. Literacy curricula compared with classrooms using High Scope or Creative Curriculum showed few differences. Both studies revealed more extensive findings using a QTE analysis applied to the *ex post* distribution of achievement than the subgroup analyses of baseline skills. Furthermore, these studies both found support for the compensatory hypothesis, finding evidence that children at the bottom of the outcome distributions benefited most. However, neither study examined SE curricula or SE outcomes, which is the objective of our study.

### Theorizing Heterogeneous Effects of Curricula Interventions

Because the findings from the SE curricular intervention literature are somewhat mixed, and only one prior distributional analysis of preschool curricula exists (Nguyen, submitted), it is unclear what a distributional analysis of SE curricula may reveal. To guide our study, we draw upon three hypotheses from the child development literature: compensatory, skills beget skills, and Goldilocks (Rutter, 1987; Cunha and Heckman, 2007; Miller et al., 2014; Nguyen, submitted).

The “compensatory” hypothesis predicts that skill-specific curricula will boost achievement for children at the bottom of the skills distribution (Rutter, 1987). In this scenario, children with the weakest skills gain the largest benefits from the treatment curricula. The “skills beget skills” hypothesis predicts that the treatment will improve children’s SE development differentially more for children in the top tier of the skills distribution (Cunha and Heckman, 2007). Here, it is the children with the strongest SE skills who are best able to take advantage of the intervention compared with children with weaker skills and, as a result, the gap between children at the low and high ends of the distribution widen due to the curricula intervention.

The “Goldilocks” hypothesis suggests that curriculum interventions will be more effective for children at the middle of the distribution, as opposed to either end of the distribution, who presumably receive just the right amount of instruction that they need from the intervention to see the largest gains. This was supported by Miller et al. (2014), who found that children with a moderate level of support from parental academic stimulation experienced the largest gains in literacy by attending Head Start. Children with lower SE skills may not benefit as much because there may be too much advanced information to learn, while children with stronger skills may not experience any benefit from the treatment because they may already possess strong skills. We also acknowledge that these hypotheses may work simultaneously.

## Current Study

We use the HS CARES study to examine children’s SE skills across children’s skill distributions. Because most Head Start children are vulnerable to low SE skills and most business-as-usual curricula do not explicitly address such unique needs, it is important to understand how curricula interventions impact children’s targeted skills along the distribution, and not solely at the mean, assuming constant treatment effects for all children. Specifically, we employ QTE to examine whether the weak treatment effects found in HS CARES vary along the distribution of several SE outcome measures, which includes direct assessments of children’s emotion, social problem-solving, and executive function skills, all of which are considered strong measures of SE development in early childhood (Campbell et al., 2016; Halle and Darling-Churchill, 2016). These outcomes were directly targeted by all three curricula interventions and possess the most reliable psychometric properties of the measures included in the impact study (Morris et al., 2014). We add to the growing literature on the distributional effects of educational programs and particularly the effects of SE skill-specific curricula for preschool programs operating at-scale. Importantly, we provide the first evidence for the effects of curricula interventions on children’s SE outcomes using QTE methodology.

## Materials and Methods

### Data

The HS CARES study is a randomized control trial conducted with childcare centers across the United States to improve child SE outcomes and includes four groups: (1) IY, (2) the PATHS, (3) Tools, and (4) the control group (i.e., business as usual). All three curricular enhancements implemented in the study were based on theory and previous research on student socio-emotional development (Raver et al., 2011; Morris et al., 2014).^1^ IY provides teacher training focusing on teachers’ management of the classroom and of children’s behavior, PATHS trains teachers to use structured lessons to help children learn about emotions and interact with peers appropriately, and Tools provides teacher training on promoting children’s learning through structured “make-believe” play, helping to strengthen children’s abilities to regulate their emotions and behavior. Implementation fidelity was examined by the impact study, finding an average fidelity score of 3.47 on a scale of 1 (low) to 5 (high), according to the surveys of coaches and trainers (Morris et al., 2014).^2^ We consider all three treatments as one treatment, comparing it with the control group (center *N* = 104).

Centers with similar racial/ethnic composition and program characteristics (e.g., full-day vs. part-day) were grouped in a single block. Centers were then randomly assigned to one of the three treatment groups or to the control group within blocks.^3^ Eighteen out of 22 blocks included four centers, and four blocks included five centers. For example, if there are four centers in a block, one center would be assigned to IY, one would be assigned to PATHS, one would be assigned to Tools, and one would be assigned to control. An average of 2.95 classrooms and an average of nine children per classroom were included in centers (Morris et al., 2014).

### Participants

The HS CARES study collected data through direct child assessment, teacher surveys, parent surveys, and class observations. The sample includes 307 classrooms and 3,603 children (2,670 2-year-old children and 933 3-year-old children). At the child level, student skills were assessed directly by observers and by teachers on academic and socio-emotional skills as well as executive functions in the fall and spring of preschool. At the classroom level, there are detailed measures on (1) classroom observations, (2) teacher training attendance, (3) training coach information, and (4) teachers’ views of the curriculum enhancement.

Our analysis sample includes students who were not missing spring outcome assessments, ranging from 1,759 to 1,827 children per outcome analysis, for a total of 2,610 children included in our study. Table 1 presents sample characteristics for children in the full analysis sample, and then separately by treatment and control group, reflecting the greatest number of cases included in our analyses. Half of the children were female, 45 percent of the children were Hispanic, 34 percent were Black, and 26 percent of children spoke Spanish at home. The *p*-values in the last column of Table 1 demonstrate that treatment and control groups were balanced on observable characteristics and baseline skills.

**TABLE 1:** Summary statistics.

	Full sample		Treatment		Control		*p*-values
			
Outcome measures	Mean	*SD*	Mean	*SD*	Mean	*SD*	
***Emotion knowledge***							
Emotions Identification	0.73	0.22	0.74	0.22	0.71	0.22	
Emotions situations	0.50	0.18	0.50	0.18	0.48	0.19	
***Social problem-solving***							
Competent responses	1.53	1.25	1.54	1.28	1.51	1.18	
Aggressive responses	1.00	1.24	0.98	1.22	1.04	1.33	
***Executive function***							
Pencil tap	0.67	0.32	0.67	0.32	0.67	0.32	
Head to toes	3.91	4.25	3.84	4.25	4.13	4.25	

**Controls at baseline**							

Child age (months)	49.18	6.72	49.47	6.27	49.3	6.85	0.89
Female	0.50		0.49		0.52		0.65
Hispanic	0.19		0.45		0.18		0.84
Black	0.27		0.33		0.27		0.93
Other	0.21		0.06		0.2		0.44
Speak Spanish	0.04		0.26		0.05		0.97
Emotions identification	0.60	0.20	0.60	0.20	0.61	0.20	0.97
Emotions situations	0.38	0.14	0.38	0.13	0.38	0.14	0.79
Competent responses	1.42	0.92	1.4	0.89	1.5	1.01	0.07
Aggressive responses	1.03	0.96	1.04	0.95	0.99	1.00	0.40
Pencil tap	0.44	0.35	0.43	0.35	0.47	0.36	0.21
Head to toes	2.18	3.46	2.12	3.41	2.36	3.64	0.20
Student *N*	2,610		1,991		619		
Center *N*	104		78		26		

*The table includes all the variables used in the study by sample groups. The first and second columns show the mean and SD for the full analysis sample, the third and fourth columns show the same information for treatment sample, and the fifth and sixth columns indicate the information for the control group. The final column shows the *p*-values from regressions comparing characteristics of treatment student with control students. Some outcome measures were missing, so the cases for these variables are less than the analysis sample of 2,610. Since each outcome measure had different missingness, the analysis sample did not omit these missing cases.*

### Measures

The study uses child SE skills assessed during the fall and spring of preschool (Morris et al., 2014). The key outcome measures include direct assessments (which eliminate teachers’ bias) of children’s emotion knowledge, social problem-solving skills, and executive function.

#### Emotion Knowledge

Facial emotion identification and emotional situation tasks were included to assess children’s knowledge of emotions (Ribordy et al., 1988). In the emotion identification task, children were asked to label the emotions on four pictures showing happy, angry, sad, and scared expressions by pointing to one of the four pictures. For example, when the assessor asks the child to identify a happy emotion, the child is expected to point to the happy facial expression. A total of 16 items are sequentially presented to children (the order of facial expressions is different for each page), with a total of four items for each emotion. The final score is the proportion of answers that are correct. In the emotional situation task, children were asked to label the emotion of the protagonist in a story. Children listened to 16 stories describing characters in emotionally evocative situations and, after each story, were presented with a page of four facial expressions, again showing happy, angry, sad, and scared faces. For each story, children were asked to point to the expression that best represented how they felt about the story. A total of 16 stories were presented — 4 stories for each emotion. The final score is the proportion of answers that were correct.

#### Social Problem-Solving Skills

Problem-solving skills were assessed using the challenging situations task (CST) (Denham and Bouril, 1994). In the CST task, children were presented with pictures of four peer scenarios (a peer knocking down the focal child’s blocks, a peer hitting the focal child, the focal child entering a group, and a peer taking a ball from the focal child). The stories focus on peer entry and peer provocation, both challenging situations likely to elicit an emotional response from young children. After each scenario, children were asked what they would do in the situation. Two of the scenarios required open-ended responses. The open-ended responses were coded as competent (appropriately asserting oneself or calmly negotiating a solution), or aggressive (responding with verbal or physical antagonism, intimidation, or force). Each open-ended situation allowed for four possible responses; the child’s first response was coded for up to two clauses, and then the assessor asks what else the child would do, and that response is also coded for up to two clauses. The child had a total of 10 opportunities to provide an aggressive response and 10 opportunities to provide a competent response. For competent and aggressive responses, the number of responses in each category was summed so that the resulting variables range from 0 to 10.

#### Executive Function Skills

Two tasks were included to assess children’s executive function skills: Head-to-Toes (Cameron Ponitz et al., 2008) and Pencil Taps (Diamond and Taylor, 1996). The Head-to Toes task assesses children’s working memory and inhibitory control skills. Children play a game in which an independent assessor instructs them to touch their head when she directs them to touch their toes, and then to touch their toes when she directs them to touch their head. Children were scored on the number of trials they answer correctly out of 10 total trials. In the Pencil Tap task, children were asked by an independent assessor to tap on a table twice with a pencil when they tap once, and once when they tap twice, assessing the child’s working memory and inhibitory control skills. Children were scored on the proportion of trials they answered correctly out of 16 total trials. This task was included in efficacy trials with low-income preschool children, such as Head Start REDI and the CSRP, and has been shown to be predictive of executive function and academic ability (Bierman et al., 2008b; Watts et al., 2018).

#### Covariates

Shown in Table 1 are the 15 child and family covariates we include in our study. Information is included regarding child age, gender, ethnicity, language, and baseline skills (e.g., BPI, learning behaviors, emotion knowledge, problem-solving skills, and social-behavioral skills in fall preschool). Child background information was collected in parent surveys, and baseline skills were from direct assessments in classroom observations and teacher-reported skills in teacher surveys.

#### Missing Data

Our analysis sample is 2,610 children out of the total HS CARES impact study sample of 2,670 because we only include children with outcome measures at the end of preschool. For each QTE model, our analysis sample is 1,759 for social problem-solving skills measures, 1,794 for emotion knowledge measures, and 1,827 for executive functions measures. Most covariates had less than 1% missing. The missingness rates for fall scores are about 2%. Missing data in fall scores were handled by the dummy variable approach (i.e., setting missing values for the covariates and baseline achievement measures to the mean of the variable and adding a dummy variable into the prediction equations for each covariate and baseline achievement indicating whether the variable was missing). We tested the differences between children with missing measures and those without by treatment status. We did not find any differences across the measures of child characteristics and skill measures in the fall by treatment status (see Appendix Table 1).

### Analyses

We estimate QTEs to examine the effect of SE skill-specific preschool curriculum interventions on the distribution of student achievement (Firpo, 2007). QTE allows for unconditional comparisons of the achievement distributions of treatment and non-treatment students and provides heterogeneous treatment effects on the treated sample other than mean differences between treatment and control groups. In the context of experimental data, QTE is estimated by calculating the difference in the two marginal distributions of the treatment and control groups (cumulative distribution functions, or CDFs) and are identified at each quantile in a logic analogous to average treatment effects under the potential outcomes framework (Bitler et al., 2014). We pool all the data for our analyses, comparing the children in all treatment centers (*n* = 78) with children in the control centers (*n* = 26), within random assignment blocks. The three curricula treatments are considered as one to have enough power for analyses. Each student *i* has two potential outcomes: *Y_1*i*_* and *Y_0*i*_* (in the current setting, SE outcomes). Student *i* has outcome *Y_1*i*_* if assigned to the treatment group and outcome *Y_0*i*_* if assigned to the control group. *D(i)* denotes the group that student *i* is assigned to in one of the three treatment groups. If student *i* is assigned to the treatment group, then *D(i)* = 1, and if student *i* is assigned to the control group, then *D(i)* = 0. The treatment effect on student *i* is defined as *d*_*i*_ = *Y_1*i*_* – *Y_0*i*_*. The intuition of QTE is as follows. Let *Y* be a random variable with a cumulative distribution function (CDF) *F(y)*, where *F(y)* = *Pr*[*Y* < = *y*]. Then, the *q*th quantile of the distribution *F(y)* is defined as the smallest value *y*_*q*_ such that *F(y_*q*_)* is at least as large as *q* (e.g., y_0.5_ is the median). Now consider two (marginal) distributions *F*_1_ (the CDF for the potential outcomes if *D* = 1) and F_0_ (the CDF for the potential outcomes if *D* = 0). The difference between the *q*th quantiles of these two distributions is defined as *y_*q*_* = *y_*q*1_ – y_*q*0_*, where *y*_*qd*_ is the *q*th quantile of distribution *F*_*d*_. The joint distribution of (*Y_0*i*_, Y_1*i*_*) is not identified without assumptions. However, if the treatment assignment is independent of the potential outcomes, then the average treatment effect, *d* = *E*[*d*_*i*_] = *E*[*Y*_1_] *- E*[*Y*_0_], is identified. As with the average treatment effect, the randomization of the treatment implies identification of the marginal quantiles *y*_*qd*_, and thus identification of the differences in their quantiles, *y_*q*_* = *y_*q*1_ – y_*q*0_*. In the current case, the QTE is the estimate of this difference in the quantiles of the two marginal distributions. The difference between these two distributions is tested at various percentiles of SE outcomes.

**FIGURE 1:**
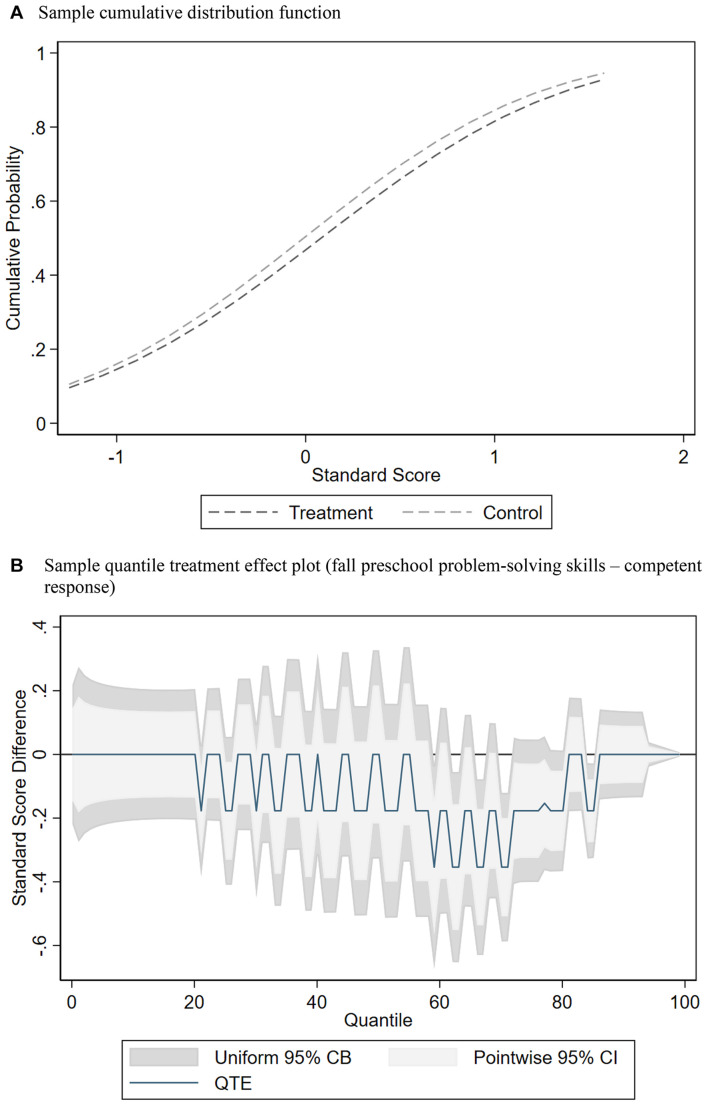
Example of randomization imbalance in the CARES study for comparison of socio-emotional curricula classrooms compared with business-as-usual classrooms on social problem-solving skills (competent response) standard scores at baseline (fall). **(A)** Sample cumulative distribution function. **(B)** Sample quantile treatment effect plot (fall preschool problem-solving skills – competent response).

Figure 1 shows an example of CDF and QTE plots for the standardized problem-solving skills for the treatment curricula classrooms compared with business-as-usual classrooms. The CDFs present the problem-solving skills on the *x*-axis with the cumulative percent of the sample on the *y*-axis. By comparing student outcomes at every single quantile from the 1st to 99th percentiles, the observed treatment effects are the horizontal differences between two CDFs at a given quantile of interest. The horizontal distance between these CDFs at each point in the distribution equals the difference in standard scores, which is the QTE at that percentile. Figure 1B shows the corresponding QTE plot, where the *x*-axis represents the cumulative percentiles of the distribution, and the *y*-axis represents the difference in standard scores between treatment and control classrooms at each percentile. The score difference (blue line) is plotted along with pointwise 95 percent confidence intervals (light gray area) and 95 percent uniform confidence bands (darker gray area), which are calculated by treatment status and bootstrapping the estimates 500 times. These plots can help to assess the degree to which randomization successfully balanced fall scores across the distribution of achievement on SE skills.

The QTE estimates are statistically significant when the two shaded gray areas, representing 95 percent confidence intervals (both as pointwise confidence intervals in light gray, and as uniform confidence bands in darker gray), do not include the horizontal black line marking zero. Pointwise confidence intervals refer to a single point estimation in each outcome between the treatment and control groups. They are narrower than a uniform confidence band, which is supposed to hold simultaneously across points. All our estimates were produced by tests for pointwise hypotheses with pointwise confidence intervals and for a functional hypothesis with uniform confidence bands, which cover the whole function with a 95% probability.^4^ Valid functional confidence bands cover the true function with the prespecified coverage probability, which can avoid issues of multiple hypothesis testing, while the union of the pointwise confidence intervals might hide the true function. In other words, while conducting a naive approach consisting of estimating many quantile regressions, pointwise tests will suffer from the multiple testing problem. Although we provide both pointwise confidence intervals and uniform confidence bands,^5^ we focus on interpreting the pointwise confidence intervals for simplicity. From a substantive perspective, we do not focus on interpreting stand-alone differences in a single percentile and think about the differences with respect to our hypotheses, in examining trends at the upper, middle, or lower ends of the distributions.

**FIGURE 2:**
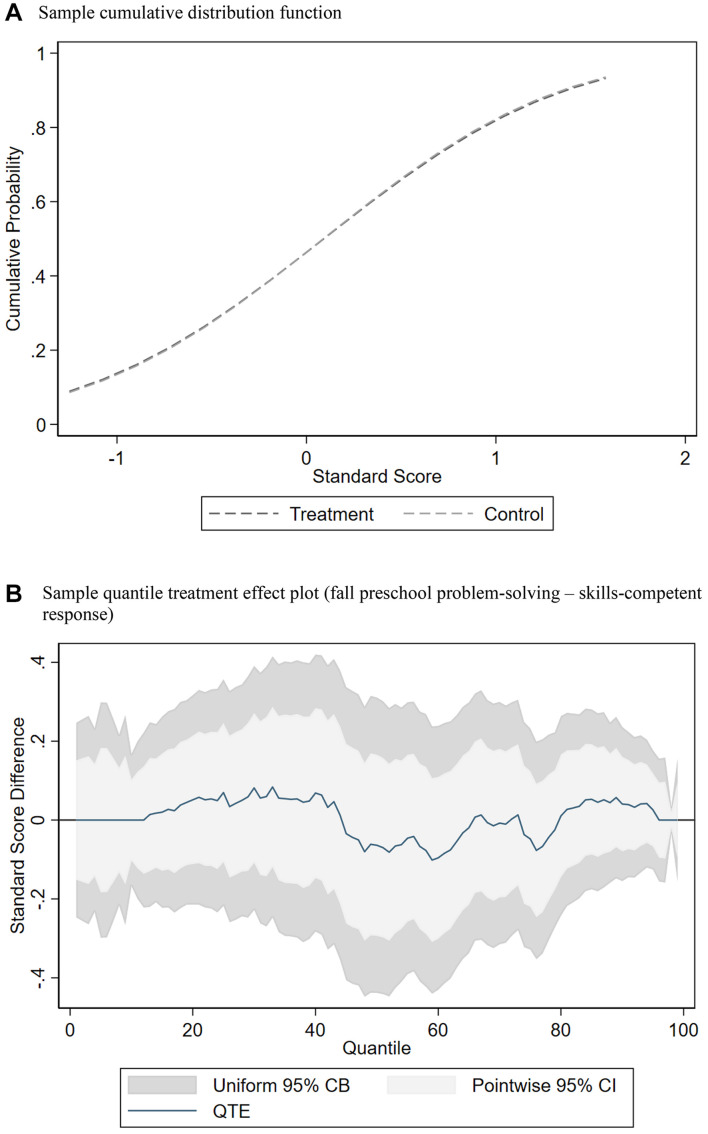
Example of balance across treatment and control achieved from inverse propensity score weighting of distributions for comparison of socio-emotional curricula classrooms compared with business-as-usual classrooms on social problem-solving skills (competent response) standard scores at baseline (fall). **(A)** Sample cumulative distribution function. **(B)** Sample quantile treatment effect plot (fall preschool problem-solving – skills-competent response).

The QTE plot in Figure 1B shows that children assigned to the control group scored higher on the problem-solving baseline measure. Between the 48th and 50th, the 70th and 76th, the 83rd and 86th, and the 89th and 95th percentiles, there is a negative and significant difference between treatment and control where the confidence intervals do not include zero, suggesting imbalance across the distribution in random assignment. We use an inverse propensity score weighting approach to balance baseline test scores between the treatment and control groups at each percentile using the set of covariates shown in Table 1 (Firpo, 2007). Propensity score weighting also allows us to account for differences in observable characteristics without examining conditional distributions. We use the unconditional QTE estimation to predict results because the QTE parameters differ in models that control for covariates compared to those that do not, which is often the case with non-linear regression models (Firpo, 2007). As shown in Figure 2, weighting creates balance because the adjusted CDF plots between the treatment and control groups are similar, and the corresponding adjusted QTE plot shows non-significance at every quantile. That is, all pointwise confidence intervals now include zero^6^.

We apply inverse propensity score weights in estimating the unconditional QTEs for measures of children’s SE outcomes. Using these balanced CDFs from the weighting procedure, the difference between these two distributions is examined at various percentiles of the outcomes. As an example, the QTE at the 50th percentile (or 0.50 quantile) can be estimated by subtracting the median test score of children in the control classrooms from the median test score of children in the treatment classrooms. By comparing test scores at a number of quantiles, we observe the effect of SE curricula on different portions of the distribution. If SE curricula have different effects for children who score relatively high, average, or low on the SE measures, this method will identify these differences, whereas mean comparisons with OLS regression would not.

## Results

**FIGURE 3:**
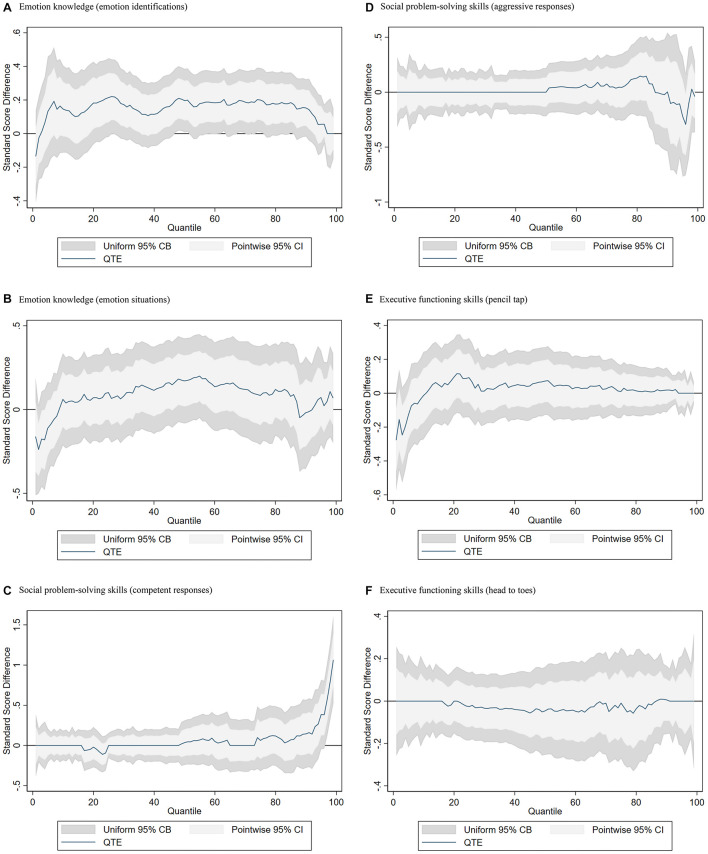
Quantile treatment effect plots at the end of preschool. Inverse propensity-score weighted quantile treatment effect of assignment to socio-emotional curricula interventions on spring preschool outcomes, SE vs. non-SE. Each graph shows a student outcome QTE for the effect of being assigned to SE Head Start centers classroom. Estimates are weighted using inverse propensity-score weights. Weights are 1/^p for treatment observations and 1/(1-^p) for control observations, where ^p is generated from a logistic regression of treatment status on baseline demographics, sample design variables, and baseline test scores. Ninety-five percent pointwise CIs and uniform CBs are shown. **(A)** Emotion knowledge (emotion identifications). **(B)** Emotion knowledge (emotion situations). **(C)** Social problem-solving skills (competent responses). **(D)** Social problem-solving skills (aggressive responses). **(E)** Executive functioning skills (pencil tap). **(F)** Executive functioning skills (head to toes).

Shown in Figures 3A–F are the distributional estimates of the test score differences (*y*-axis) between children in the treatment and control classrooms for each percentile of the distribution of the outcome variable (*x*-axis). The blue line represents the point estimate at a given quantile. When the estimate is above zero, children in treatment classrooms are outscoring children in control classrooms. Similarly, when the blue line is below zero, children in treatment classrooms are scoring lower than children in control classrooms.

### Emotion Knowledge

Figures 3A,B show the QTE plots for children’s emotion knowledge (emotion identification and emotion situation) measured in the spring. For emotion identification, the point estimates are positive, with the exception of the 1st and 2nd percentiles and the 97th to 99th percentiles. Between the 45th and 90th percentiles, the point estimates are statistically significant, with the largest point estimate of 0.21, although some estimates (e.g., 78th percentile) are insignificant in terms of uniform confidence bands. This pattern of results shows significant positive effects of the SE intervention for children with relatively strong emotional identification skills, between the 45th and 90th percentiles in outcome scores, controlling for all baseline characteristics and test scores using inverse propensity score weights (see Table 1 for details).

The emotion situation results are similar, but the estimates are smaller and not statistically significant. Most of the estimates are positive, but from the 1st to 9th percentiles and the 88th to 91st percentiles, the point estimates are negative.

### Social Problem-Solving

Figures 3C,D show the QTE plots for problem-solving skills: competent responses and aggressive responses. For competent responses, the point estimates are close to zero and slightly positive, except for the 17th to 24th percentiles, but all insignificant. From the 95th to 99th percentiles, the point estimates are positive and statistically significant, with the largest point estimate of 1.14 at the 99th percentile. This means that children at the very top of the competent response score distribution who were exposed to a SE curriculum improved their social problem-solving skills by as large as 1.14 SD, controlling for all baseline characteristics and test scores.

For aggressive responses, the point estimates are primarily near zero, apart from the estimates after the 50th percentile. From the 51st to 85th percentiles, the point estimates are positive, but insignificant. From the 86th to 99th percentiles, except for the 98th percentile, the point estimates are negative and insignificant.

### Executive Function

Figures 3E,F show the QTE plots for children’s executive function skills, including the measures of pencil tap and head-to-toes. For the pencil tap, the point estimates are primarily near zero and positive, with the exception of the negative estimates between the 1st and 10th percentiles. All point estimates are statistically insignificant, controlling for all baseline characteristics and test scores. For the head-to-toes measure, the point estimates are also near zero, with some exceptions of negative and positive estimates across the score distributions, but all estimates are statistically insignificant.

## Discussion

Understanding the heterogeneity of treatment effects for different children is important for unpacking the modest, and sometimes null effects of educational interventions. Educational and developmental researchers most commonly test for this by examining how well distinct subgroups of children are performing on an outcome. Our study complements the traditional subgroup analyses of heterogeneity, which examine treatment effects by children’s baseline characteristics with QTE. QTE analyses permit us to evaluate the effects of an intervention at various parts of the outcome distribution, including the upper and lower tails, which could unpack hidden impacts after random assignment to understand who benefits most from the SE curricula (Schochet et al., 2014). This methodology can provide a more comprehensive understanding of variation in treatment effects, including those previously hidden by the standard average impacts estimated in the original Head Start CARES evaluation (Morris et al., 2014). We tested three different hypotheses of heterogeneous treatment effects of curricula interventions compared with business-as-usual curricula on children’s SE skills. Our goal was to provide distributional evidence on children’s SE development, which is a particularly important and policy-relevant issue in Head Start programs, as children in these settings are more likely to experience SE difficulties (Yoshikawa et al., 2012).

Consistent with our hypotheses, we find positive impacts of curricula interventions on two SE skills, but not equally across the full distribution of children’s skills. We observe a “skills beget skills” effect in that children at the higher end of the social problem-solving skills distribution, measured by the competent response CST, benefit the most from the treatment curricula (Heckman, 2006). This implies that children with the strongest SE skills are best able to take advantage of the intervention compared with children with weaker skills. We also find a “Goldilocks” effect for children in the higher middle of emotion knowledge distributions, measured by the facial emotions task for facial emotion identification. Here, it is possible that children at the higher-middle of the distribution, having the right amount of such skills to acquire emotional knowledge, are better able to piece together different emotions more accurately (Miller et al., 2016).

Both of these patterns are consistent with, and complementary to, the positive mean findings from CARES’ impact study (Morris et al., 2014). It is possible that the average effects picked up by the impact study are because children at the highest end of the distribution experience the largest gains on social problem-solving skills, or because children in the higher middle of the emotion knowledge distribution benefited, in our “Goldilocks” finding. The effects of average group differences are of greatest relevance in intervention evaluations, but these evaluations assume that children at the top and bottom of the distribution are experiencing identical processes throughout the course of the intervention. This is not an incorrect assumption in evaluations if most students benefit from the intervention. However, we found that for social problem-solving skills, the processes affecting children at the higher end of the skills distribution are not the same as those affecting children at the bottom of the distribution. Children with higher social problem-solving skills in the treatment group may have received the right instruction to develop their skills.

Our findings for social problem-solving skills and emotional knowledge derived from QTE also complement previous subgroup analysis findings, because QTE analyses can more precisely capture SE curricula *ex post* effects on specific outcome measure while linear regression results for subgroup analysis based on baseline skills capture their *ex ante* effects. For example, the impact study found that children in the lowest quartile of academic skills at baseline and from “high-risk” households benefited more from curricula interventions in terms of socio-emotional outcomes (Morris et al., 2014). Distinct from their findings, we found that children at the higher end of social problem-solving skills and in the higher middle of emotional knowledge skills outcomes distributions benefited most from the SE curricula intervention. Specifically, we observed a “skills beget skills” effect in those children at the 95th to 99th percentiles of social problem-solving skills and “Goldilocks” effect for children between 45th and 90th percentiles of emotional knowledge skills benefited most. These results were not captured in simple subgroup analyses likely because of the reordering of posttests from the intervention, and imbalance in pretests between treatment and control groups in their skill distributions.

Our QTE findings of SE intervention effects on the (*ex post*) distribution are of substantial interest for closing achievement gaps between rich and poor children, and can therefore offer practical insights. Our findings for social problem-solving and emotional knowledge skills were not detected in the impact study, suggesting that additional supports for teachers may be beneficial during implementation, such as helping teachers to work with children with lower skills (as we found that children with lower social problem-solving and emotional knowledge skills did not benefit from the SE curricula). Our findings further suggest that practices for children with stronger SE skills may further enhance the impacts of the intervention. Our finding that children at the bottom of the skill distribution did not benefit from the SE curricula also suggests that the SE curricula interventions alone are insufficient for supporting the most socially and emotionally struggling children, and that teachers should consider ways to address such instruction. Given Head Start’s mission of serving young children “at risk” of academic or developmental challenges, particular attention should be paid to the resources and practices to support such children.

These findings echo the need for interventions to consider both the characteristics of the program and the individual needs of the child (Duncan and Vandell, 2012). Ideal directions for practice include interventions designed to consider children’s individual differences and offer specific and individualized instructional practices directed to children’s needs within these domain-focused curricula. However, this raises issues in the alignment between children’s early learning experiences and their assessed skills. One suggestion for practice would be to encourage teachers to tailor instruction for each child on the basis of their problem-solving and emotional knowledge skills across a variety of instructional contexts in the classroom, such as creating a positive class climate, providing additional emotional support, and scaffolding children to regulate their emotions and behaviors. In practice, however, tailoring each student’s need based on their SE development is a hard task for teachers in everyday classrooms, particularly when the funding for such programs is constrained or with larger class sizes. Thus, teachers should be supported in these areas, given that positive teacher–child and peer–peer interactions are productive tools for teachers to provide differentiated and individualized instruction (Pianta et al., 2017). With guidance and support from curricular materials or coaches, teachers can incorporate into their planning additional explicit direct instruction of SE content or adapt their instructional strategies and activities more flexibly to optimize children’s learning based on their needs in the moment-to-moment context of instructional opportunities. Such practices are particularly critical for children with lower skills, as we did not find children with lower emotion knowledge and social problem-solving skills benefited from the SE interventions. Future SE interventions might need to offer more support or consider adding more effective features of programs to help children with lower SE skills.

Aside from the positive findings for emotional knowledge and social problem-solving skills, we did not find significant effects at any points in the outcome distributions for executive function skills, aggressive problem-solving skills, or emotion situation knowledge, consistent with the null results on these skills from CARES’ impact study and thus ruling out possible hidden treatment effects across the distribution of skills (Morris et al., 2014). Indeed, one of the most important conditions of conducting a distributional analysis is that the interventions produce positive effects or that hidden effects have not been studied before (Jackson and Page, 2013). These results also echo the insignificant findings from previous studies using QTE to evaluate the effects of early interventions on children’s non-cognitive outcomes (Bitler et al., 2014) and null findings on SE skills from SE curricula (Baron et al., 2017).

While SE interventions could improve children’s SE skills, it may be more challenging to improve their executive function skills. Children’s EF skills were not improved from the SE interventions in both this study and the evaluation study, which explicitly targeted children’s behavior regulation and EF skills (e.g., The Incredible Years and Tools of the Mind—Play). This might be because the measurement of these outcomes is not widely tested and there is a lack of precision in measuring those outcomes, although the measures used in CARES’ study were recommended in most prior studies (Morris et al., 2014). However, it may not be accurate to conclude that SE curricula interventions do not improve children’s executive functions. One reason is that the domains encompassed in SE skills are not independent from each other, and previous research found that emotion and executive functions are interrelated (Carlson and Wang, 2007; Ferrier et al., 2014). Additionally, previous studies did not evaluate SE curricula on multiple SE skills (e.g., studies only examined EF as opposed to other SE skills such as emotion knowledge). Another explanation for the lack of EF findings is that we only examined EF skills at the preschool level; EF are higher-level cognitive skills which usually develop with age (Best and Miller, 2010). This logic could hold true for emotion situation knowledge and aggressive problem-solving skills as well. While children are still learning to identify emotions or respond to tasks, it might be difficult for them to develop more advanced skills, such as situating an emotion or triggering an aggressive response. Future interventions and evaluations about EF development are needed to identify approaches to assess and support children’s development in this area.

Socio-emotional development is gaining greater attention in the early childhood field, as evidence shows that children’s early SE skills can provide a foundation for their school readiness and success, particularly for low-income children (Bierman et al., 2008a; Diamond, 2013, 2016; Morris et al., 2014; Lee et al., 2016). It is critical to assess whether early childhood interventions targeting SE skills are having their desired effects of boosting children’s SE skills while narrowing achievement gaps. To justify implementing a SE curriculum, we will also need to know whether they generate cross-domain impacts on other essential school readiness outcomes such as cognitive skills. Future work could use a similar approach to understanding variation in treatment effects for other curricula or programs, such as those targeting children’s math skills. Answering these questions will be key for understanding who benefits most from these increasingly popular early childhood curriculum-based interventions.

## Data Availability Statement

The data analyzed in this study is subject to the following licenses/restrictions: The dataset is restricted to researchers who apply to use the data offline. Requests to access these datasets should be made via https://www.childandfamilydataarchive.org/cfda/archives/cfda/studies/35510/summary.

## Ethics Statement

Ethical review and approval was not required for the study on human participants in accordance with the local legislation and institutional requirements. Written informed consent to participate in this study was provided by the participants’ legal guardian/next of kin.

## Author Contributions

ZS was responsible for the preparation of the study, data preparation and analysis, and the writing of the current manuscript. JJ supported the study design and gave feedback on the data analysis and the current manuscript on multiple occasions. Both authors contributed to the article and approved the submitted version.

## Conflict of Interest

The authors declare that the research was conducted in the absence of any commercial or financial relationships that could be construed as a potential conflict of interest.

## Publisher’s Note

All claims expressed in this article are solely those of the authors and do not necessarily represent those of their affiliated organizations, or those of the publisher, the editors and the reviewers. Any product that may be evaluated in this article, or claim that may be made by its manufacturer, is not guaranteed or endorsed by the publisher.

## Footnotes

1 While the three SE curricula were implemented on top of base curricula which varied across centers, center characteristics were similar. Specifically, like a typical Head Start center, the majority (nearly 70%) of centers used Creative Curriculum. The rest of the centers reported that High/Scope, a state-adapted core curriculum, or DLM Early Childhood Express served as their primary classroom curricula (Morris et al., 2014).

2 Specifically, the fidelity scores for IY, PATHS, and Tools were 3.69, 3.73, and 2.97 respectively, which are considered satisfactory.

3 Seventeen grantees were selected to participate in the study located in 10 states across the nation. Grantees were recruited in two phases with two cohorts. Cohort One consisted of 4 grantees in the Northeast and participated during the 2009–2010 school year, while Cohort Two consisted of 13 grantees from the rest of the country and participated during the 2010–2011 school year. There was a total of 104 centers within 22 blocks included across 17 grantees in the study, including nine grantees with 4 participating centers each (i.e., a total of 36 centers), seven grantees with 8 participating centers each (i.e., a total of 56 centers), and one grantee with 12 participating centers.

4 We used the Stata command “qrprocess,” which provides functional confidence bands and tests of functional hypotheses using a multiplier bootstrap of 500 (Chernozhukov et al., 2020).

5 The results for both pointwise confidence interval and uniform confidence bands are consistent.

6 Note that the balance checks between the original study and our study are different. While the HS CARES study assessed balance in the means of children’s characteristics and skills between treatment and control centers, our study assesses balance in children’s skills across distributions from the 1st to 99th percentiles between the treatment and control centers. This is consistent with other studies, finding differences in these two distinct checks (Bitler et al., 2014; Penner, 2016). The baseline balance check is essentially conducted in the same way as our main analysis, using QTE for baseline skill distributions, finding differences in baseline skills across skill distributions. This type of diagnostic is another advantage of using QTE.

## Appendix

**APPENDIX TABLE 1:** Test of differences in fall variables missingness by treatment status.

	Selected child characteristics					Fall skill measures					
		
						Emotion knowledge		Problem-solving skills		Executive function	
	Female	Speak Spanish	Hispanic	Black	Other	Emotion identification	Emotion situation	Competent response	Aggressive response	Pencil tap	Head to toes
Assigned to treatment	0.00	–0.01	–0.00	–0.00	–0.00	–0.02	–0.03	–0.02	–0.02	–0.01	–0.01
	(0.00)	(0.05)	(0.05)	(0.05)	(0.05)	(0.05)	(0.05)	(0.05)	(0.05)	(0.05)	(0.05)
*N*	2,610	2,610	2,610	2,610	2,610	2,610	2,610	2,610	2,610	2,610	2,610

*Robust standard errors were adjusted for clustering at center level shown in parentheses. All missing indicators were created based on the analysis sample of 2,610. All models were run separately for each measure.*
